# The competitive advantage of sanctioning institutions revisited: A multilab replication

**DOI:** 10.1093/pnasnexus/pgad091

**Published:** 2023-05-02

**Authors:** Sergio Lo Iacono, Wojtek Przepiorka, Vincent Buskens, Rense Corten, Marcel van Assen, Arnout van de Rijt

**Affiliations:** Department of Sociology, University of Essex, Colchester CO4 3SQ, UK; Department of Sociology/ICS, Utrecht University, Utrecht 3584 CH, The Netherlands; Department of Sociology/ICS, Utrecht University, Utrecht 3584 CH, The Netherlands; Department of Sociology/ICS, Utrecht University, Utrecht 3584 CH, The Netherlands; Department of Sociology/ICS, Utrecht University, Utrecht 3584 CH, The Netherlands; Department of Sociology/ICS, Utrecht University, Utrecht 3584 CH, The Netherlands; Department of Methodology and Statistics, Tilburg University, Tilburg 5037 AB, The Netherlands; Department of Political and Social Sciences, European University Institute, Fiesole FI 50014, Italy

## Abstract

Is peer sanctioning a sustainable solution to the problem of human cooperation? We conducted an exact multilab replication (*N* = 1,008; 7 labs × 12 groups × 12 participants) of an experiment by Gürerk, Irlenbusch, and Rockenbach published in *Science* in 2006 (Gürerk Ö, Irlenbusch B, Rockenbach B. The competitive advantage of sanctioning institutions. 2006. Science. 312(5770):108–111). In GIR2006 (*N* = 84; 1 lab × 7 groups × 12 participants), groups that allowed members to reward cooperators and punish defectors were found to outgrow and outperform groups without a peer-sanctioning institution. We find GIR2006 replicated in accordance with all preregistered replication criteria in five of the seven labs we sampled. There, the majority of participants joined groups with a sanctioning institution, and participants cooperated and profited more on average than in groups without a sanctioning institution. In the two other labs, results were weaker but still favored sanctioning institutions. These findings establish the competitive advantage of sanctioning institutions as a robust phenomenon within the European context.

**Significance Statement**

When individual and collective interests are in conflict, individuals face a social dilemma whereby cooperation will lead to higher collective benefits but acting selfishly will produce higher individual benefits. Oftentimes, individuals facing such dilemmas may join and subject themselves to institutions that promote cooperation. This paper probes the sustainability of peer-sanctioning institutions in fostering cooperative behaviors. Under peer sanctioning, if sufficient individuals are willing to incur the costs of punishing uncooperative behaviors, then, cooperators fare better than noncooperators. However, peer sanctioning can effectively promote cooperation only if groups that practice it can thrive in competition with groups that do not. Our replication study shows that, across different populations, groups with peer sanctioning consistently outgrow and outperform groups without peer sanctioning.

## Introduction

The problem of cooperation is encountered in countless instances where individuals or organizations must collaborate to achieve a desired goal (1, 2). Many of the world's most existential challenges are cases where cooperation has failed. Present-day governments face the momentous task of convincing citizens and businesses to reduce fossil fuel use, while simultaneously finding themselves in a cooperation problem with other nations. Research on the evolution of cooperation has informed public policy at the highest level, with leading scientists serving on committees advising governments on coping with nuclear crises, pandemics, and climate change. It is therefore no exaggeration to claim that major advances in the field of human cooperation can have global impact.

In many circumstances, actors facing cooperation problems may join and subject themselves to institutional setups that promote cooperation. Buyers and sellers may elect to operate on online sales platforms that implement peer-rating systems enabling trust in anonymous transactions (3) or collaborate on platforms that use symbolic awards to compel contributors to volunteer work (4). Countries that voluntarily join the International Criminal Court (ICC) or the United Nations Framework Convention on Climate Change subject themselves to negative sanctions upon the violation of agreements. Many countries' ambivalent stance towards the ICC and the US government's temporary withdrawal from the Paris Agreement show how fragile these institutions are and how important it is to understand the conditions under which they are formed and can be maintained for the benefit of all.

The present paper probes the sustainability of one of the most prominent mechanisms put forward for providing incentives for cooperative behavior: peer sanctioning (5). Under peer sanctioning, if sufficient individuals are willing to incur the costs of rewarding cooperators or punishing defectors, then cooperators fare better than defectors (6, 7). It has been shown that particularly peer punishment of defectors promotes cooperation in social dilemmas (3, 8). However, peer sanctioning can only be a solution to the puzzle of human cooperation, if groups that practice it can thrive in competition with groups that do not (9–11). Are sanctioning institutions (SI) able to sustain an equilibrium in which individuals that volunteer to incur the costs of sanctioning profit more than individuals in sanctioning-free institutions (SFI)?

This question of sustainability of SI was first addressed in an experimental study by Gürerk and colleagues published in *Science* in 2006 (hereafter GIR2006) (12). GIR2006 let participants choose whether to interact in a public goods dilemma in an environment with or without a SI (13). They found that almost all participants migrated to the environment with a SI. Moreover, the SI was effective in maintaining cooperation and yielded higher payoffs than the SFI. However, GIR2006's important findings were based on an experiment with 84 participants clustered in 7 groups at a single lab.

Following GIR2006, several studies have investigated the choice between SI and SFI in public goods games (PGG). For instance, Sutter et al. (14) and Markussen et al. (15) explored how voting affects institutional choices among different sanctioning environments, showing that self-organization may yield a positive impact on cooperation levels. Such valuable contributions, however, tend to deviate considerably from GIR2006 (e.g., in terms of institutional environments available, group size, or number of periods). Gürerk et al. (16) and Gürdal et al. (17) conducted experiments with designs closer to GIR2006, corroborating the main findings of the original study in Germany and Turkey albeit with some variations in parameter settings and the participation of authors of the original study.

In light of the current replication crisis in the social sciences (18–20), we provide a comprehensive assessment of GIR2006's findings by reporting results from replications of the original study in seven different lab locations (see Materials and methods). We chose these labs based on their suitability to (i) recreate the conditions of the original study and (ii) test the robustness of the original findings across participant pools different from the one of the original study. Because of the COVID-19 pandemic, we were forced to cancel experiments in three labs, but managed to conduct experiments in two new, unplanned labs (see Materials and methods). Each replication had 144 participants clustered in 12 groups (*N* = 1,008 participants in total), achieving greater statistical power than GIR2006 at each lab. Multilab replications like ours offer a more definitive assessment of replicability of effects, showing whether the study's results can be generalized across different participant pools (21–23), and are slowly becoming more standard in several disciplines (e.g. psychology; 24).

To accurately evaluate whether effects reported in GIR2006 replicate, we conducted an exact replication; changing the experimental design or testing new hypotheses was therefore precluded. Our replication follows Brandt et al.'s criteria for replicating an experimental study (25). The effects intended for replication were carefully defined before data collection, preregistered, and critically compared to the results of the original study. We obtained all materials to conduct the experiments and analyze the data from the authors of the original article (i.e. z-Tree code, instructions and protocol, stimuli, measures, procedures, and analyses; see Materials and methods and Supplementary material, Section A, Table S1, Section C, and Figs. S1–S8). All details of the replication were verified by the corresponding author of GIR2006. The complete details about the replication are available on the Open Science Framework (OSF) website, where we preregistered the replication, the analysis plan, stored the data, and the statistical analysis code (https://osf.io/tyxfm/?view_only=5f560d5d570241eaa460b094aed074b4).

In GIR2006, participants interacted anonymously in groups of 12 for 30 rounds. Each round, each participant had to make two or three choices. First, participants decided whether they wanted a SFI or a SI. Second, participants decided how much of their individual endowments [i.e. 20 monetary units (MUs)] they wanted to contribute to a public good (i.e. a standard linear PGG; see Materials and methods). Third, all participants received 20 additional MUs, but only those in the SI could decide to reward or punish their fellow group members at a cost to themselves after receiving feedback about these group members’ contributions. Finally, at the end of a round, participants were informed on all group members’ contributions, sanctions and rewards received, and earnings.

We predefined three replication criteria that must be jointly met in order to consider GIR2006 replicated in a given lab (see preregistration on OSF): (1A) in significantly more than 50% of groups, a majority chooses SI in the final round, and at least 75% of participants choose SI in the final round; (1B) the average contributions and (1C) average profit are significantly higher in SI than in SFI in the final round. As GIR2006, we used the Wilcoxon signed-rank matched pairs test with groups as observations and a significance threshold of *α* = 0.05 for two-sided tests. To increase statistical power, we increased the number of groups at each lab from 7 (original study) to 12, maintaining a total of 12 participants in each group (see Materials and methods).

## Materials and methods

### Experimental design

**Fig. 1.**
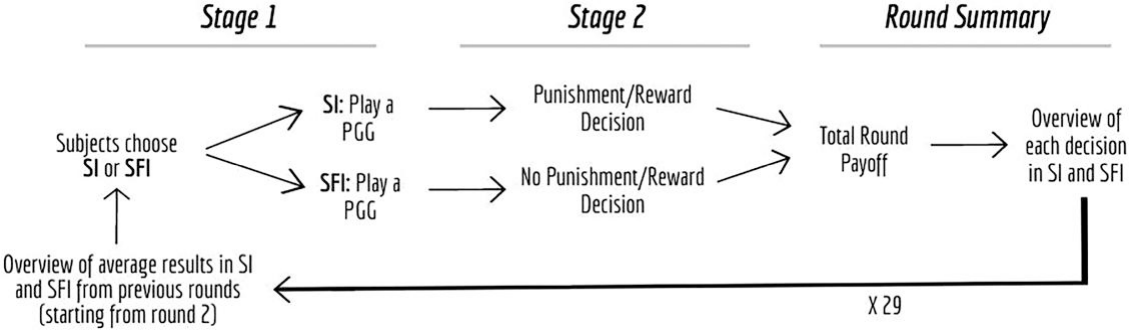
Overview of GIR2006 experimental design.

As in GIR2006, in our replications, participants interact anonymously in groups of 12 over 30 rounds. At the beginning of the experiment, participants are assigned an endowment of 1,000 tokens to minimize the risk of bankruptcy. Each round consists of two stages (Fig. 1).

In stage 1, participants can choose which institutional condition they want to join: a SFI, where participants cannot exercise influence on the earnings of other group members, or a SI, where participants can exercise influence on the earnings of other group members by assigning positive or negative tokens. After choosing the institutional condition, participants play a standard linear PGG. Participants are assigned an endowment of 20 tokens and decide whether they want to invest their tokens in a collective project or keep the tokens for themselves (i.e. their contribution). The sum of all tokens invested in the collective project by all group members within their institutional condition (i.e. either SFI or SI) is multiplied by 1.6 and redistributed evenly among group members. A participant *i*'s payoff $p_{i,1}$ from stage 1 is calculated as follows:


$$p_{i,1}=20-c_{i}+\frac{1.6}{n_{e}}\;\overset{n_{e}}{\underset{\;j=1}{\sum }}\;c_{j},$$

where $c_{i}$ is participant *i*'s contribution, *e* is the institutional condition chosen by participant *i*, and $n_{e}\;$ is the number of participants in the institutional condition *e*.

In stage 2, participants can see other group members’ contributions to the collective project in their institutional condition (i.e. either SFI or SI). As the order of presentation is randomized, it is not possible to identify other players by their position on the list across rounds. In this stage, each participant is assigned an endowment of 20 tokens. Participants in SI need to decide whether they want to assign tokens to increase or reduce the payoff of other group members in their institutional condition or to keep it unchanged by not assigning tokens. Positive tokens increase the payoff of other participants at a ratio of 1:1 (i.e. one positive token increases the payoff of another participant by one token), while negative tokens decrease the payoff of other participants at a ratio of 1:3 (i.e. one negative token decreases the payoff of another participant by three tokens). Participants themselves lose the tokens they assigned to increase or decrease the payoff of another participant. Participant *i*'s payoff $p_{i,2}$ from stage 2 is calculated as follows:


$$p_{i,2}=\{\begin{array}{c} 20-s_{i}+\;r_{i}^{+}-3r_{i}^{-},\;\;\;\;\;\;\;\mathrm{if}\;e=\mathrm{SI},\\ 20,\;\;\;\;\;\;\;\;\;\;\;\;\;\;\;\;\;\;\;\;\;\;\;\;\;\;\;\;\;\;\;\;\;\;\;\;\mathrm{if}\;e=\mathrm{SFI}, \end{array}$$

where $s_{i}$ is the number of (positive and negative) tokens participant *i* assigned to other group members, $\;r_{i}^{+}$ is the number of positive tokens participant *i* received, and $r_{i}^{-}$ is the number of negative tokens participant *i* received.

At the end of the round, the total round payoff for each participant is computed and shown to the participant. The total round payoff is the sum of the participant's payoff from stages 1 and 2, $p_{i,\mathrm{tot}}=\;p_{i,1}+\;p_{i,2}$. Note that if a participant is the only group member in an institutional condition, he/she will earn 20 tokens from stage 1 and 20 tokens from stage 2 but he/she will not be able to make any decision. Finally, each participant receives an overview of the results in both SI and SFI. For every group member, participants are informed about contribution to the collective project, payoff from stage 1, assigned tokens (if applicable), received tokens (if applicable), and total round payoff. Once again, as the order in which information about the other players is presented is randomized in each round, it is not possible to identify other players by their position on the list across rounds. Starting with round 2, in stage 1, before deciding which institutional condition they want to join, participants receive an overview of the average results of all previous rounds at the bottom of the screen. They can see the following information: average contribution of members of SFI and SI, average payoff for members of SFI and SI, average positive tokens received for members of SI, and average negative tokens received for members of SI.

### Data collection

Data were collected between 2019 October 10 and 2021 July 16 in seven labs, all located in different countries: Italy (Bologna), Germany (Nuremberg), the United Kingdom (Oxford), The Netherlands (Utrecht), Spain (Valencia), Poland (Warsaw), and Switzerland (Zurich)—see Table 1. We obtained all materials to conduct the experiment from the authors of the original article. This allowed us to use the very same z-Tree code, instructions, and protocol as employed by GIR2006. However, we translated the material from German to English, Spanish, Italian, and Polish, following as closely as possible the original study. All translated materials were double-checked by two independent researchers that were proficient in the respective language and made available on OSF.

**Table 1.** Overview of data collection.

Lab	Location	Fieldwork dates	Data collection	Language	Before/after beginning of COVID-19	z-Tree version
BLESS	Bologna (IT)	2020 November 12–2020 November 18	In-lab	Italian	After	4.1.9
LEARN	Nuremberg (DE)	2021 June 22–2021 July 16	Online^a^	German	After	5.1.3
CESS	Oxford (United Kingdom)	2019 November 12–2019 November 14	In-lab	English	Before	3.6.6
ELSE	Utrecht (NL)	2019 October 10–2020 January 21	In-lab	English	Before	4.1.9
LINEEX	Valencia (ES)	2020 March 3–2020 March 5	In-lab	Spanish	Before	4.1.9
LEE	Warsaw (PL)	2019 December 10–2020 January 17	In-lab	Polish	Before	4.1.9
ETH DeSciL	Zurich (CH)	2019 November 5–2019 November 28	In-lab	German	Before	4.1.9

a Online data collection via z-Tree unleashed (26).

In line with GIR2006, we recruited participants at each location using a standard sample of the lab population. Participants at each location were paid in compliance with the average hourly pay rate and show-up fees employed at the lab. Replications were closely supervised across all labs by the same researcher who instructed lab assistants on the experiment's protocol to minimize as much as possible deviations from the original study. We recruited 144 participants at each location, reaching a total sample size of 1,008 participants.

Our original plan for data collection included labs from the United States (New York), Chile (Santiago de Chile), the United Arab Emirates (Abu Dhabi), the United Kingdom (Oxford), The Netherlands (Utrecht), Spain (Valencia), Poland (Warsaw), and Switzerland (Zurich). However, we had to change our data collection plans because of the COVID-19 pandemic outbreak. We therefore focused on labs that were allowed to stay open while complying with applicable COVID-19 norms, ensuring the safety of lab participants. This led us to drop the labs in New York, Santiago de Chile, and Abu Dhabi, and include the labs in Bologna and Nuremberg instead.

Data collection in Bologna occurred in-lab and complied with the GIR2006 protocol within the limits imposed by the applicable COVID-19 norms (e.g. wearing face masks and 1.5-m distance rules). In Nuremberg, we had to deviate more substantially from the GIR2006 protocol as we collected data online via z-Tree unleashed using the lab sample pool. To mimic as much as possible in-lab conditions, each session in Nuremberg was supervised via chat by a lab assistant who could support participants in case they had a question or needed help. Once the minimum number of participants were connected to the online session and confirmed their identity, the lab assistant started the experiment. According to our protocol, a participant was considered as dropped out in the online sessions if (i) his/her timeout occurred for more than 3 min, (ii) he/she did not respond to chat messages for 3 times after the timeout occurred, and (iii) he/she did not move forward with the experiment. If a participant dropped out during a session (either online or in-lab), the session was considered failed, and observations were discarded accordingly. No participant dropped out during any of our sessions (either online or in-lab). If not enough participants showed up for a session, the experiment did not take place and participants were paid their show-up fee and invited to participate in another session. Across all replications, only two sessions failed after the start of the experiment because of technical issues (one in Zurich and one in Nuremberg).

### Sample size considerations

GIR2006 tested their hypotheses using the Wilcoxon signed-rank matched pairs test with groups as observations. However, the power of this test is rather low with seven groups (in the original study); the null hypothesis fails to be rejected if the sum of negative ranks equals three or more. GIR2006 found higher scores in SI than in SFI in all groups, yielding a sum of negative ranks of 0, leading them to reject the null hypothesis despite the low power of the test. But if SFI had scored higher than SI in just one of GIR2006's seven groups, and the absolute difference in scores in that one deviating group had been greater than the margins by which SI outperformed SFI in at least two of the other groups, then they would have failed to reject the null hypothesis.

For this reason, we decided to increase statistical power by increasing the number of groups for each replication from 7 to 12, keeping 12 subjects in each group. For 12 groups, the null hypotheses for results 1A–1C are rejected if the sum of signed ranks is 13 or less, which means the null hypotheses may still be rejected if 4 out of 12 groups have a majority (1A) or higher scores (1B and 1C) in SFI than in SI. In this manner, we are much more likely to corroborate 1A–1C if the effects observed by GIR2006 do exist (see Supplementary material, Section A, for further considerations on power analysis). Note that replication criterion 1A consists of two parts, the second part being that at least 75% of the participants choose SI. This second part was added to prevent that 1A is corroborated with the Wilcoxon test without a large majority of individuals in SI averaged across all groups. We selected the 75% threshold in respect to the proportion of respondents who choose SI because it represents an overwhelming majority while at the same time is lenient with regard to the original finding of 92.9%.

## Results

Replication results for 1A–1C are shown in Table 2. *In five labs, replication criteria 1A, 1B, and 1C were jointly satisfied, thereby replicating GIR2006's findings* (Bologna, Nuremberg, Utrecht, Valencia, and Zurich): in the final round in more than 50% of groups, a majority chose SI and at least 75% of participants chose SI; average contributions and profits were higher in SI than in SFI. GIR2006's findings were not replicated in Oxford and Warsaw; only 63.9% of participants chose SI in Oxford in the last round, and in both Oxford and Warsaw, the average earnings were not statistically significantly higher than those in SFI.

**Table 2.** Behaviors in the last round for each lab.

Replication criteria	1A % choosing SI or SFI		1B Contribution (MUs)		1C Profit (MUs)	
	Mean (SI)	Mean (SFI)	Mean (SI)	Mean (SFI)	Mean (SI)	Mean (SFI)
	Wilcoxon *z* value		Wilcoxon *z* value		Wilcoxon *z* value	
GIR2006 *N*_g_ = 7; *N*_i_ = 84	92.86%	7.14%	19.29	3.21	51.39	41.93
	2.40*		2.39*		2.37*	
Bologna^a^ *N*_g_ = 12; *N*_i_ = 144	81.25%	18.75%	16.12	2.99	48.03	41.79
	2.72**		3.06**		3.06**	
Nuremberg^a,b^ *N*_g_ = 12; *N*_i_ = 144	88.19%	11.81%	18.86	3.40	50.58	42.04
	3.10**		3.06**		3.06**	
Oxford *N*_g_ = 12; *N*_i_ = 144	63.89%	36.11%	14.36	2.18	42.59	41.31
	1.73		3.06**		0.71	
Utrecht N_g_ = 12; N_i_ = 144	91.67%	8.33%	18.19	2.14	49.06	41.28
	3.12**		3.06**		2.83**	
Valencia *N*_g_ = 12; *N*_i_ = 144	91.67%	8.33%	10.67	1.29	45.29	40.78
	3.09**		2.98**		2.59**	
Warsaw *N*_g_ = 12; *N*_i_ = 144	83.33%	16.67%	18.02	2.37	45.79	41.42
	2.88**		3.06**		1.26	
Zurich *N*_g_ = 12; *N*_i_ = 144	96.53%	3.47%	19.76	1.96	50.07	41.17
	3.17**		3.07**		3.06**	

*N*_g_ and *N*_i_ indicate the sample size for each lab at the group and individual levels, respectively. *Replication criteria*: in the final round, (*1A*) in significantly more than 50% of groups, a majority chooses SI and at least 75% of participants choose SI; (*1B*) the average contributions are significantly higher in SI than in SFI in the final round; and (*1C*) the average profit is significantly higher in SI than in SFI in the final round. Behaviors in the last round replicate GIR2006's results in Bologna, Nuremberg, Utrecht, Valencia, and Zurich but not in Oxford and Warsaw.

a Data collection occurred after the beginning of the COVID-19 pandemic.

b Online data collection via z-Tree unleashed (26).

MUs, monetary units; SI, sanctioning institution; SFI, sanctioning-free institution.

*P ≤ 0.05, **P ≤ 0.01, and ***P ≤ 0.001 (for two-sided tests).

**Fig. 2.**
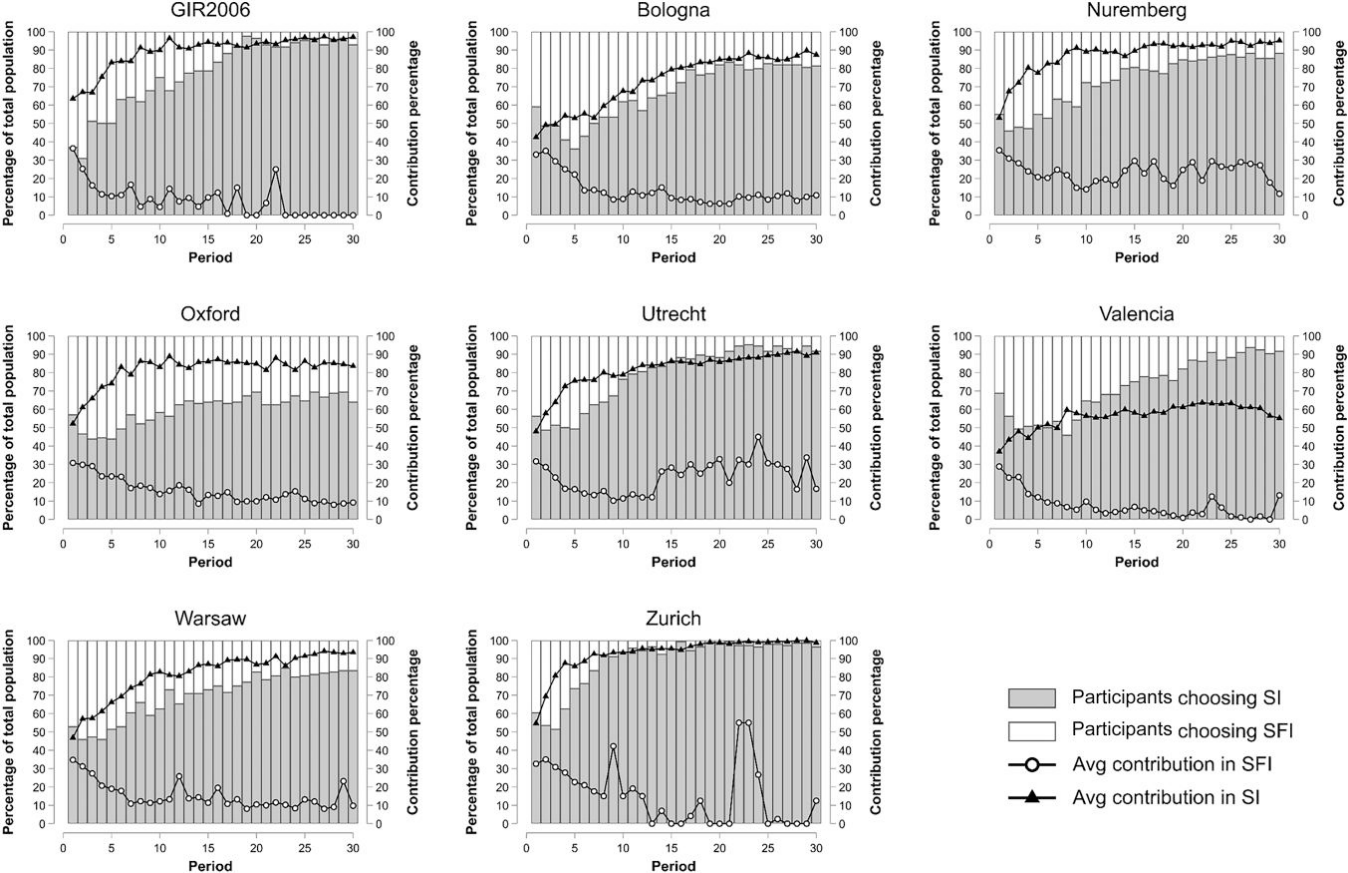
Migration choices and contributions across rounds at each lab. SI, sanctioning institution; SFI, sanctioning-free institution.

**Fig. 3.**
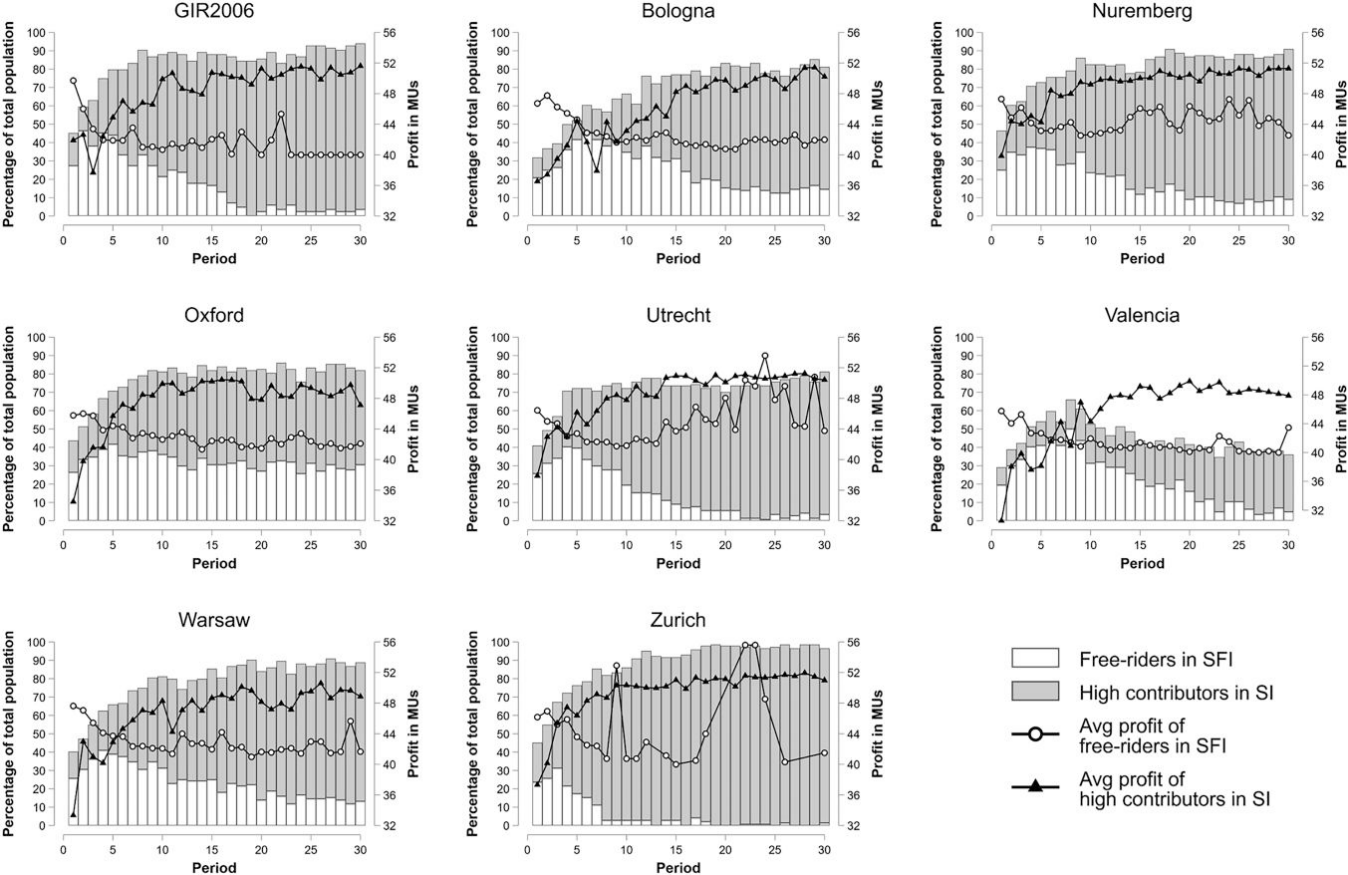
Payoffs and incidence of high contributors in SI and free riders in SFI across rounds at each lab. MUs, monetary units; SI, sanctioning institution; SFI, sanctioning-free institution. Participants are defined as free riders if they contributed between 0 and 5 MUs and as high contributors if they contributed between 15 and 20 MUs.

Findings for 1A–1C are the product of a dynamic process triggered by participants’ behavior across rounds. To put these results in context, we examined participants’ behaviors in the first round and across rounds in the same way GIR2006 did (Table 3 and Figs. 2 and 3; see also Table S2). In doing so, we critically evaluate how key findings from GIR2006 other than the ones preregistered as replication criteria compare with results from each of the labs.

**Table 3.** Behaviors in the first round and across rounds for each lab.

	Panel A—behaviors in the first round						Panel B—behaviors across rounds					
	% choosing SI or SFI		Contribution (MUs)		Profit (MUs)		Effect of punishment on PGG contribution	% high contributors who punish	Profit before round 5 (MUs)		Profit after round 5 (MUs)	
	Mean (SI)	Mean (SFI)	Mean (SI)	Mean (SFI)	Mean (SI)	Mean (SFI)	Tobit coefficient	Mean (SI)	Mean (SI)	Mean (SFI)	Mean (SI)	Mean (SFI)
	Wilcoxon *z* value		Wilcoxon *z* value		Wilcoxon *z* value				Wilcoxon *z* value		Wilcoxon *z* value	
GIR2006 *N*_g_ = 7; *N*_i_ = 84	36.90%	63.10%	12.96	7.17	39.20	44.30	0.444 (0.085)***	22.21%	36.70	42.57	47.14	41.37
	−2.16*		2.37*		−2.03*				−2.92**		3.46***	
Bologna^a^ *N*_g_ = 12; *N*_i_ = 144	59.03%	40.97%	8.64	6.42	35.75	43.85	0.468 (0.046)***	29.56%	35.90	43.71	44.16	41.43
	1.82		2.51*		−3.06**				−5.67***		3.70***	
Nuremberg^a,b^ *N*_g_ = 12; *N*_i_ = 144	54.86%	45.14%	11.18	7.34	36.33	44.64	0.525 (0.097)***	19.91%	40.31	43.73	48.02	42.91
	1.03		1.87		−2.49*				−2.87**		9.05***	
Oxford *N*_g_ = 12; *N*_i_ = 144	56.94%	43.06%	10.87	5.95	33.14	43.57	0.312 (0.052)***	22.47%	36.65	43.37	44.93	41.98
	1.63		2.90**		−2.98**				−4.63***		5.73***	
Utrecht *N*_g_ = 12; *N*_i_ = 144	56.25%	43.75%	9.30	6.63	35.33	43.98	0.522 (0.105)***	18.54%	38.15	42.99	47.49	42.46
	1.41		1.80		−3.06**				−4.32***		7.04***	
Valencia *N*_g_ = 12; *N*_i_ = 144	68.75%	31.25%	7.36	5.81	34.73	43.48	0.642 (0.085)***	29.06%	36.11	42.81	43.09	40.81
	3.05**		1.69		−2.93**				−5.24***		1.35	
Warsaw *N*_g_ = 12; *N*_i_ = 144	52.78%	47.22%	9.18	7.00	33.12	44.20	0.407 (0.053)***	25.12%	36.10	43.44	44.72	41.64
	0.68		1.02		−3.06**				−4.82***		3.83***	
Zurich *N*_g_ = 12; *N*_i_ = 144	60.42%	39.58%	10.99	6.41	33.87	43.85	0.497 (0.075)***	15.85%	39.28	43.75	49.62	42.46
	2.09*		2.90**		−3.06**				−3.46***		5.43***	

*N*_g_ and *N*_i_ indicate the sample size for each lab at the group and individual levels, respectively. Behaviors in the first round mimicked irregularly the initial conditions of GIR2006, except for profit levels (Panel A). Yet, game dynamics are similar across labs, replicating the patterns observed in GIR2006 (Panel B). As GIR2006, we used a Tobit regression model to assess the effect of punishment (i.e. negative sanctions in round *t*) on PGG contribution (i.e. contribution*_t_*_+ 1_ − contribution*_t_*) for subjects who chose SI in round *t* and *t* + 1, controlling for positive sanctions in round *t* and estimated robust SEs (in parentheses) accounting for clustering within each group of 12 participants.

a Data collection occurred after the beginning of the COVID-19 pandemic.

b Online data collection via z-Tree unleashed (26).

MUs, monetary units; SI, sanctioning institution; SFI, sanctioning-free institution.

*P ≤ 0.05, **P ≤ 0.01, and ***P ≤ 0.001 (for two-sided tests).

Table 3 panel A shows that GIR2006's findings for the first round were only partially replicated. In all labs, in line with GIR2006, the average profit in the first round was higher in SFI than SI. However, while in GIR2006, participants in the first round showed a stronger preference for SFI than for SI, in our locations, participants either did not have a stronger preference for SFI (in Bologna, Nuremberg, Oxford, Utrecht, and Warsaw) or actually preferred SI over SFI (in Zurich and Valencia). Also, as in GIR2006, contribution levels were significantly higher in SI than SFI in Bologna, Nuremberg, Oxford, and Zurich, but not in Utrecht and Warsaw.

Yet, results in Table 3 panel B and Figs. 2 and 3 indicate that *game dynamics were similar across the different labs, replicating the patterns observed in GIR2006*: sanctioning promotes higher contributions; about 20% of high contributors in SI punish low contributors; and profit is significantly higher in SI after round 5. The only exception in this regard is Valencia, where the average contribution level is rather low and profit in SI becomes significantly higher only after round 10 (before round 10, Mean_SI_ = 37.30 MUs, Mean_SFI_ = 41.83 MUs, Wilcoxon signed-rank test, *z* = −6.063, *P* ≤ 0.001; after round 10, Mean_SI_ = 44.22 MUs, Mean_SFI_ = 40.70 MUs, Wilcoxon signed-rank test, *z* = 4.492, *P* ≤ 0.001).

## Discussion

This exact multilab replication study shows that the emergence and persistence of peer-sanctioning institutions in experimental public goods settings is a robust phenomenon. Allowing individuals to join, leave, and rejoin the institutional environment of their choice, either with or without peer sanctioning, leads to the endogenous emergence of SI that maintains cooperation, while securing higher individual profits than environments without SI. Across different subject populations from seven labs and varying starting conditions in terms of migration choices and cooperation, groups with peer sanctioning consistently outgrew and outperformed groups without peer sanctioning. Our three preregistered replication criteria were jointly met in five of the seven locations. In the remaining two locations, results were weaker than in the original study yet still favored peer sanctioning.

Our study was an exact (sometimes also called “direct” or “close”) replication study (25). In exact replication studies, the original experimental procedures are replicated as closely as possible. This necessitated the use of the same parameter settings in all trials across all locations. While this allows us to claim a robust experimental result, it at the same time raises the question of how sustainable peer sanctioning would be for other parameter values, as the exact values in the original experiment are rather arbitrary. Specifically, important questions to answer are what individual costs of sanctioning can be tolerated before the dominance of peer sanctioning breaks down, and whether larger subject populations have an easier or harder time establishing sustainable cooperation through peer sanctioning.

The experiments performed in the seven labs, nevertheless, varied along a range of dimensions (mostly outside our control): outbreak of the COVID-19 pandemic, data collection method (in-lab or online), language, time of year, and hour of the day. The corroboration of the competitive advantage of SI despite this variability suggests that the results of GIR2006 are not unique to a particular population of subjects in a specific contextual setting. However, one should be reluctant to generalize our conclusions to locations not incorporated in our study, particularly locations with different cultural traditions (27, 28). Our exact replication effort required that the same person—the lead author—served as experimentalist on-site to ensure successful procedural implementation. This rendered it difficult, especially under pandemic-related restrictions on travel and social distancing in laboratories, to repeat the experiment in distant locations. Recent studies found that variability in lab procedures was a better predictor of replication failure than cultural differences (21, 22, 29), reinforcing the importance we placed on exact replication at the expense of geographical variability. Expanding on the present contribution, future replications of GIR2006 could test if findings hold across different cultural contexts (e.g. Western vs. non-Western countries) and sample pools (e.g. convenience samples vs. representative samples of the general population).

Finally, the effort to closely replicate the original study similarly led us to consider competition only between regimes with and without peer sanctioning. A worthwhile line of inquiry is a broadening of the pool of institutional regimes that compete for dominance, including centralized sanctioning, reputation systems, communication systems, and ostracism of defectors. This would not only provide a more stringent test of the robustness of peer-sanctioning regimes as institutional form, but also allow the identification of institutional arrangements that, once offered, would be likely to emerge and sustain group cooperation in real-world settings.

## Supplementary Material

pgad091_Supplementary_Data

## Data Availability

All data and materials generated or analyzed during this study are available on the Open Science Framework (OSF) repository (https://osf.io/tyxfm/?view_only=604fe6ace95d4e52b89ec8556c4fb304).
