# Supplementary material for: The competitive advantage of sanctioning institutions revisited: A multilab replication
Source: PNAS Nexus. 2023 May 2;2(5):pgad091. doi: 10.1093/pnasnexus/pgad091 (PMC10153419; doi:10.1093/pnasnexus/pgad091)
Supplement: pgad091_Supplementary_Data [file pgad091_supplementary_data.zip › PNASNEXUS-PNASNEXUS-2022-01089-s03.rtf]

[1] -- Tuesday, January 31, 2023 -- 09:45:16
t tests - Means: Wilcoxon signed-rank test (matched pairs)
Options:	A.R.E. method
Analysis:	Sensitivity: Compute required effect size 
Input:	Tail(s)	=	Two
	Parent distribution	=	Normal
	á err prob	=	0.05
	Power (1-â err prob)	=	0.8
	Total sample size	=	12
Output:	Noncentrality parameter ä	=	3.0946217
	Critical t	=	2.2149511
	Df	=	10.4591559
	Effect size dz	=	0.9141790
