# Supplementary material for: The competitive advantage of sanctioning institutions revisited: A multilab replication
Source: PNAS Nexus. 2023 May 2;2(5):pgad091. doi: 10.1093/pnasnexus/pgad091 (PMC10153419; doi:10.1093/pnasnexus/pgad091)
Supplement: pgad091_Supplementary_Data [file pgad091_supplementary_data.zip › PNASNEXUS-PNASNEXUS-2022-01089-s01.docx]

**Supplementary Materials**

The competitive advantage of sanctioning institutions revisited: a multi-lab replication

*Section A – Materials and Methods2*

*Section B – Further Results4*

*Section C – Instructions6*

Pre-registration, replication material (in 5 languages), and datasets used in this study can be found on the Open Science Framework (OSF) website: <https://osf.io/tyxfm/?view_only=5f560d5d570241eaa460b094aed074b4>

**Section A – Materials and Methods**

*Data Analysis Plan and Measures*

We obtained all materials to reproduce the original figures and analyze the data from the authors of GIR2006. We employed the same operationalizations of concepts and statistical tests to assess behaviors in the first round and across rounds at each location. We pre-registered our data analysis plan and related Stata do.file (in Stata version 16.1) on OSF. To assess behaviors in the last round, which was not directly tested in GIR2006 but was part of our pre-registered replication criteria 1A-1C (see main text), we applied the following adjustment to analyze the data: when, in a session, one of the institutional conditions was chosen by only one or no player in the last round, we considered the values of contribution and profit from the last available round where at least two subjects chose that institutional condition.

*Demographics*

**Table S1 – Descriptive statistics**

|  | | *Mean* | *SD* | *Min* | *Max* | *N* |
| --- | --- | --- | --- | --- | --- | --- |
| Bologna | Age | 24.16 | 2.81 | 18 | 34 | 144 |
|  | Gender (Male) | 0.46 | 0.50 | 0 | 1 | 144 |
| Nuremberg | Age | 25.29 | 3.85 | 18 | 40 | 144 |
|  | Gender (Male) | 0.44 | 0.50 | 0 | 1 | 144 |
| Oxford | Age | 31.42 | 15.26 | 18 | 64 | 144 |
|  | Gender (Male) | 0.44 | 0.5 | 0 | 1 | 144 |
| Utrecht | Age | 23.98 | 6.54 | 18 | 64 | 144 |
|  | Gender (Male) | 0.33 | 047 | 0 | 1 | 144 |
| Valencia | Age | 20.69 | 2.80 | 18 | 45 | 144 |
|  | Gender (Male) | 0.45 | 0.50 | 0 | 1 | 144 |
| Warsaw | Age | 23.27 | 4.11 | 18 | 40 | 144 |
|  | Gender (Male) | 0.47 | 0.5 | 0 | 1 | 144 |
| Zurich | Age | 22.31 | 4.45 | 18 | 57 | 144 |
|  | Gender (Male) | 0.40 | 0.49 | 0 | 1 | 144 |

*Further considerations on power analysis*

In addition to null-hypothesis significance testing (NHST – see main text) we also evaluated the effect sizes on our three main outcome variables ‘choice of institution’, ‘contribution’, and ‘profit’, both in the original and the seven replication labs. Effect sizes were very large for the original study (Cohen’s d_z_’s > 3.8). Each of the seven labs had a statistical power of at least 0.8 to detect a true effect size d_z_ ≥ .914, and a power equal to 1 if true effect size d_z_ = 3.8.^[[1]](#footnote-1)^ We applied the Bayesian snapshot method (Van Aert & Van Assen, 2017) to evaluate effect sizes on the three variables at all locations, calculating posterior probabilities (p_p_) of zero, large (d_z_ = 0.8) or very large (d_z_ = 4) true effect sizes given a uniform prior. To summarize, p_p_ > .95 for a very large effect on all three variables in the original study and at Bologna, Nuremberg, and Zurich. A very large effect on ‘contribution’ was found in all labs (p_p_ = 1). Noteworthy is that for Oxford p_p_ = .99 for a large effect on contribution and p_p_ = 1.00 for a zero effect on profit, whereas Warsaw and Valencia had large p_p_ for a large effect on profit (.95 and .82, respectively). To conclude, heterogeneous effects were observed with smaller but still large effects on profit found in Warsaw and Valencia, whereas in Oxford the effect on contribution was smaller than in the original study and the effect on profit was not replicated.

**Section B – Further Results**

*Other Key Findings from GIR2006*

In addition to the key findings presented and discussed in the main text, here we further illustrate how participants behaved in the first round and across rounds, in line with what we set out in our pre-registration. Table B1, panel A shows the percentage of participants who were high contributors (i.e., those who contribute at least 75% of their endowment) in SI and SFI, and the percentage of high contributors who exercised punishment in SI in the first round. Table B1, panel B shows the percentage of participants who contributed more after migrating from SFI to SI, the percentage of participants who contributed less after moving from SI to SFI, and the percentage of high contributors in SI across all rounds. Results are consistent with our conclusions in the main text.

**Table S2 – Further results on behaviors in the first round and across rounds at each location**

|  | | Panel A - Behaviors in the first round | | | Panel B - Behaviors across rounds | | | | | | | |
| --- | --- | --- | --- | --- | --- | --- | --- | --- | --- | --- | --- | --- |
|  | | *% High contributors* | | *% High contributors who punish* | *% Contributing more after migration to SI* | *% Contributing less after migration to SFI* | | *% High contributors in SI* | | | | |
| GIR2006  (N_g_ = 7)  (N_i_ = 84) | Mean (SI) | 50.95% | | 73.34% | 81.93% | 71.55% | | 86.91% | | | | |
|  | Mean (SFI) | 10.77% | |  |  |  | |  | | | | |
|  | Wilcoxon z-value | 2.38* | |  |  |  | |  | | | | |
| Bologna^†^  (N_g_ = 12)  (N_i_ = 144) | Mean (SI) | 18.22% | | 50.00% | 74.55% | 71.70% | | 66.37% | | | | |
|  | Mean (SFI) | 8.61% | |  |  |  | |  | | | | |
|  | Wilcoxon z-value | 1.34 | |  |  |  | |  | | | | |
| Nuremberg^†‡^  (N_g_ = 12)  (N_i_ = 144) | Mean (SI) | 42.64% | | 54.84% | 74.92% | 63.01% | | 84.71% | | | | |
|  | Mean (SFI) | 19.20% | |  |  |  | |  | | | | |
|  | Wilcoxon z-value | 1.96* | |  |  |  | |  | | | | |
| Oxford  (N_g_ = 12)  (N_i_ = 144) | Mean (SI) | 33.17% | | 84.00% | 73.96% | 63.84% | | 76.11% | | | | |
|  | Mean (SFI) | 9.03% | |  |  |  | |  | | | | |
|  | Wilcoxon z-value | 2.83** | |  |  |  | |  | | | | |
| Utrecht  (N_g_ = 12)  (N_i_ = 144) | Mean (SI) | 25.70% | | 81.82% | 78.80% | 72.46% | | 72.91% | | | | |
|  | Mean (SFI) | 12.73% | |  |  |  | |  | | | | |
|  | Wilcoxon z-value | 1.62 | |  |  |  | |  | | | | |
| Valencia  (N_g_ = 12)  (N_i_ = 144) | Mean (SI) | 13.95% | | 78.57% | 77.93% | 65.61% | | 30.58% | | | | |
|  | Mean (SFI) | 10.61% | |  |  |  | |  | | | | |
|  | Wilcoxon z-value | 0.59 | |  |  |  | |  | | | | |
| Warsaw  (N_g_ = 12)  (N_i_ = 144) | Mean (SI) | 27.79% | | 76.19% | 74.45% | 62.41% | | 78.63% | | | | |
|  | Mean (SFI) | 17.45% | |  |  |  | |  | | | | |
|  | Wilcoxon z-value | 0.94 | |  |  |  | |  | | | | |
| Zurich  (N_g_ = 12)  (N_i_ = 144) | Mean (SI) | 36.16% | | 80.65% | 79.55% | 60.63% | | 92.53% | | | | |
|  | Mean (SFI) | 10.97% | |  |  |  | |  | | | | |
|  | Wilcoxon z-value | 2.55* | |  |  |  | |  | | | | |
|  |  |  |  | |  | |  | |  |  |  |  |

**Note.** N_g_ and N_i_ indicate the sample size for each lab at the group and individual level respectively. † Data collection occurred after the beginning of the COVID-19 pandemic. ‡ On-line data collection via z-Tree unleashed. * p ≤ 0.05, ** p ≤ 0.01, *** p ≤ 0.001 (for two-sided tests).

**Section C – Instructions**

Here, we provide the introduction, the instructions to the experiment, and the additional written instructions that substitute the slide presentation employed in GIR2006. The additional written instructions were verified and approved by the corresponding author of GIR2006 before data collection. The German version (from the original study), as well as the Italian, Spanish, and Polish versions of all materials necessary to replicate GIR2006 are available on OSF.

**Introduction**

Welcome to this session and thank you for taking time from your schedule to be here today.

You are participating in an experiment in which you can earn some money. How much you earn depends on the decisions you will make and the decisions other participants will make.

Your decisions and the decisions of the other participants remain anonymous and will not be linked to any information that would allow to identify you (for example your name). The results of this study serve a purely scientific purpose.

The money you earn in this session will be paid out to you in cash at the end of the session. The other participants will not be able to see how much you earned.

This session will last about 2 hours. Thus, if you need to use the restroom, please do it now.

Please note that during the session you are not allowed to communicate with the other participants, and we kindly ask you to mute or switch off your phone and store it in your bag or coat.

I will now start reading aloud the instructions that lie in front of you on the desk.

**Instructions to the Experiment**

**General information**

At the beginning of the experiment you will be randomly assigned to one of **2 subpopulations, each consisting of 12 participants**. During the whole experiment you will only interact with the members of your subpopulation.

At the beginning of the experiment, each participant is assigned an **endowment of 1,000 tokens** to their account.

**Course of action**

The experiment consists of **30 rounds**. Each round consists of 2 stages. In Stage 1, the choice of group and the decision regarding the contribution to the collective project take place. In Stage 2, participants may exercise influence on the earnings of the other group members.

**Stage 1**

**(i) The choice of group**

In Stage 1, each participant decides which group (s)he wants to join.

There are two different groups:

|  | **Exercise influence on the earnings of other group members** |
| --- | --- |
| **Group** | A: No |
|  | B: Yes, by assigning positive and negative tokens |

**(ii) Contribution to the Collective Project**

In Stage 1 of each round, each group member has an **endowment** of **20 tokens**.

You have to decide how many of the 20 tokens you are going to contribute to the collective project. The remaining tokens are yours to keep.

**Calculation of your payoff in Stage 1**

Your payoff in Stage 1 consists of two components:

- **tokens you have kept** = endowment – your contribution to the collective project
- **earnings from the project** = 1.6 x sum of the contributions of all group members / number of group members

| Thus, **your payoff in Stage 1** amounts to:  20 – your contribution to the collective project +  1.6 x sum of the contributions of all group members to the collective project / number of group members |
| --- |

The earnings from the collective project are calculated according to this formula for each group member.
**Please note:** Each group member receives the same earnings from the project, that is each group member benefits from **all** contributions to the collective project.

**Stage 2**

**Assignment of tokens**

In Stage 2 it will be displayed how many tokens each group member contributed to the collective project. (**Please note: The order of presentation is randomly determined in each round)** Thus, it is not possible to identify any group member by their position on the displayed list throughout different rounds.)

By **your assignment of tokens,** you can **increase** or **reduce** the payoff of a group member or **keep it unchanged**.

In each round each participant receives an **additional 20 tokens** in Stage 2. You have to decide how many of these 20 tokens you are going to **assign** to other group members. The remaining tokens are yours to **keep**. You can check the costs of your token assignment by pressing the button “Token calculator”.

- **Each** **positive token** that you assign to a group member **increases his/her payoff** by **1 token**.
- **Each** **negative token** that you assign to a group member **reduces his/her payoff** by **3 tokens**.
- If you assign **0 tokens** to a group member you **don’t change the payoff** of that group member.

**Calculation of your payoff in Stage 2**

Your payoff in Stage 2 consists of three components:

- **tokens you have kept** = 20 – sum of the tokens that you have assigned to the other group
   members
- **plus the number of positive tokens** you have received from other group members
- **minus the 3-fold number of negative tokens** you have received from other group members

| Thus, **your payoff in Stage 2** amounts to:  20 – sum of the tokens that you assigned to other group members  + the number of positive tokens you received from other group members  – 3 x (the number of negative tokens you received from other group members) |
| --- |

**Calculation of your round payoff**

Your round payoff is composed of:

|  | Your payoff from Stage 1 | = 20 – your contribution to the collective project   + 1.6 x (sum of all project contributions) / (number of group members) |
| --- | --- | --- |
| + | Your payoff from Stage 2 | = 20 – sum of the tokens that you assigned to other group members  + number of positive tokens you received   – 3 x (number of negative tokens you received) |
| = | Your round payoff | |

**Special case: single group member**

If it happens that you are the **only member** in your group, you receive 20 tokens in Stage 1 and 20 tokens in Stage 2, that is your round payoff sums up to 40. You have neither in Stage 1 nor in Stage 2 a possibility to take an action.

**Information at the end of the round**

At the end of the round you receive a detailed overview of the results obtained in all groups. For every group member you are informed about : contribution to the project, payoff from Stage 1, assigned tokens (if possible), received tokens (if possible), payoff from Stage 2, round payoff.

**History**

Starting with the 2^nd^ round, before the beginning of a new round, you receive an overview of the average results (as above) of all previous rounds.

**Total payoff**

The total payoff from the experiment is composed of the initial endowment of 1000 tokens plus the sum of round payoffs from all 30 rounds.

At the end of the experiment your total payoff will be paid out at an exchange rate of 1 £ per 100 tokens.

**Please note**

Communication is not allowed during the whole experiment. If you have a question please raise your hand. All decisions are made anonymously, that is no other participant is informed about the identity of someone who made a certain decision. The payment is anonymous too, that is no participant learns what the payoff of another participant is.

We wish you success!


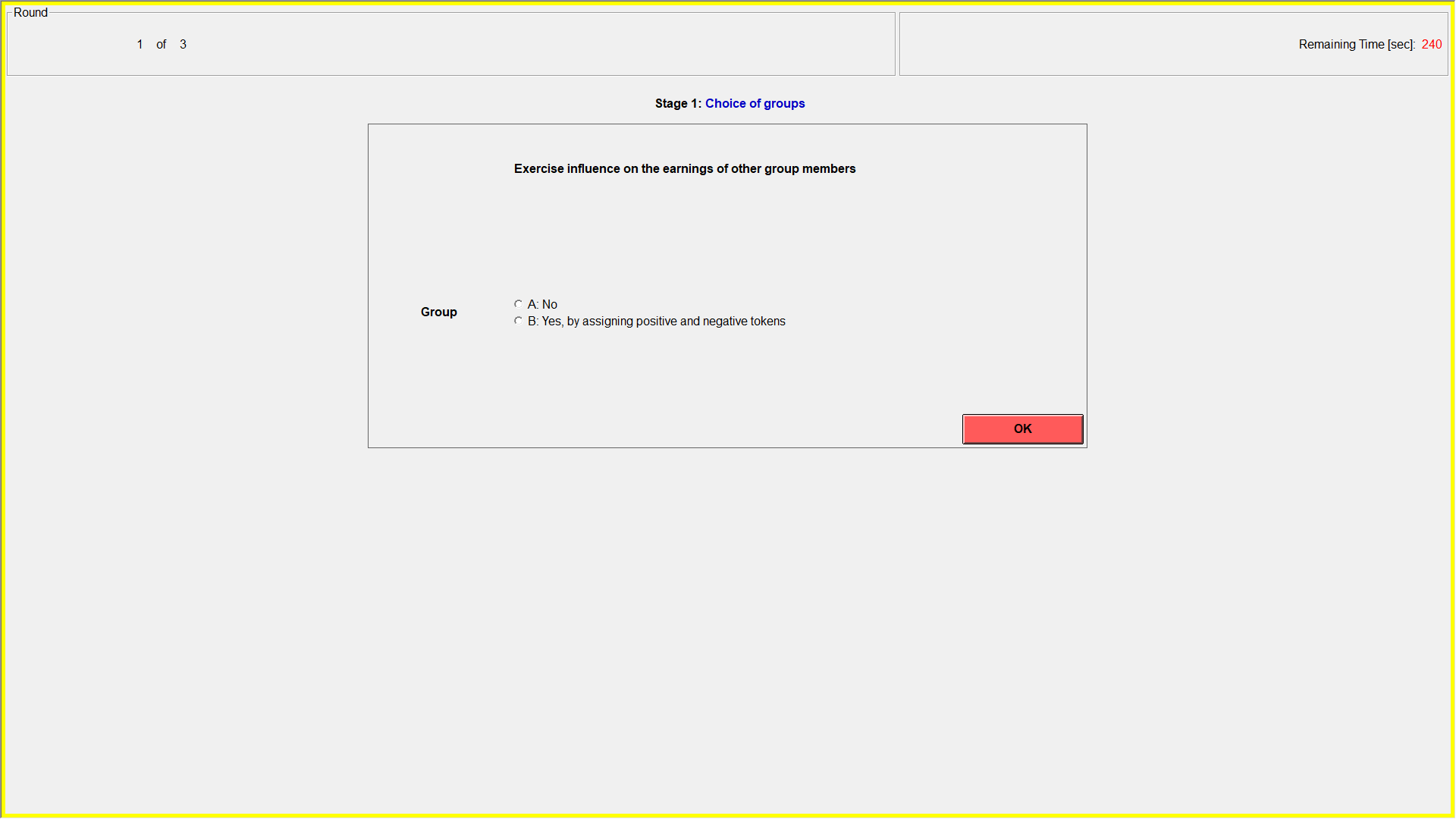
**Figure S1.**

This is the “Stage 1: Choice of group” screen. Here you can choose which group you want to join. In Group B, participants can exercise influence on the earnings of other group members by assigning positive and/or negative tokens, whereas in Group A they do not have this option.In the top-left corner you can see in which round you are. In the top-right corner you can see your remaining time to reach a decision. Note that this is an example. In the experiment, you will play **30 rounds**, not 3 rounds.


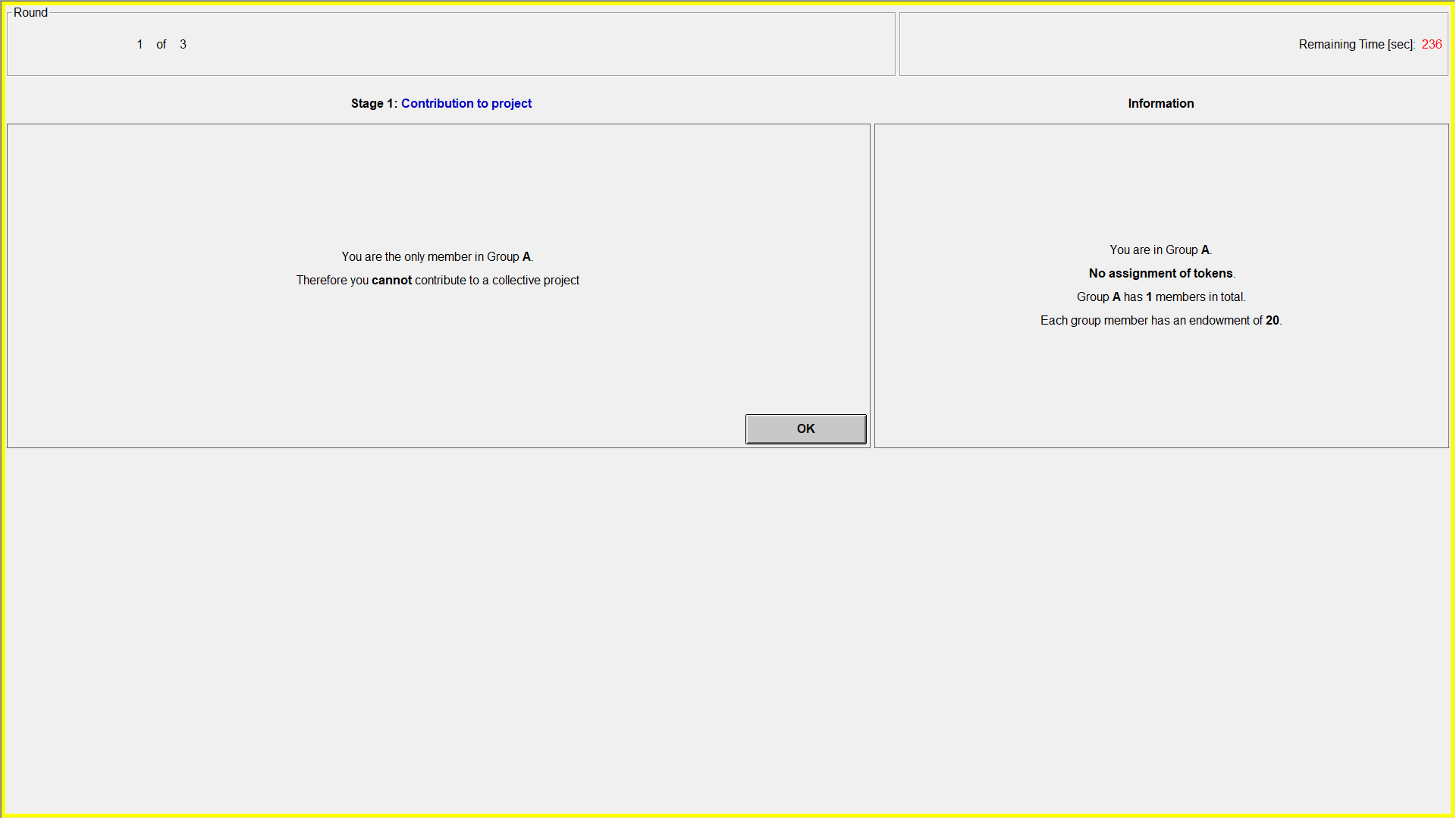
**Figure S2.**

This is the “Stage 1: Contribution to project” screen. Here you can decide how much you want to contribute to the collective project. The box on the right indicates in which group you are, the number of group members, and the endowment of each group member.

In this particular example, you are the only member in Group A. You receive 20 tokens in this stage. Because you are the only member in Group A, you have no possibility to take action in either Stage 1 or Stage 2 of the game. If there are at least two members in Group A, you can choose how much you want to contribute to the collective project.


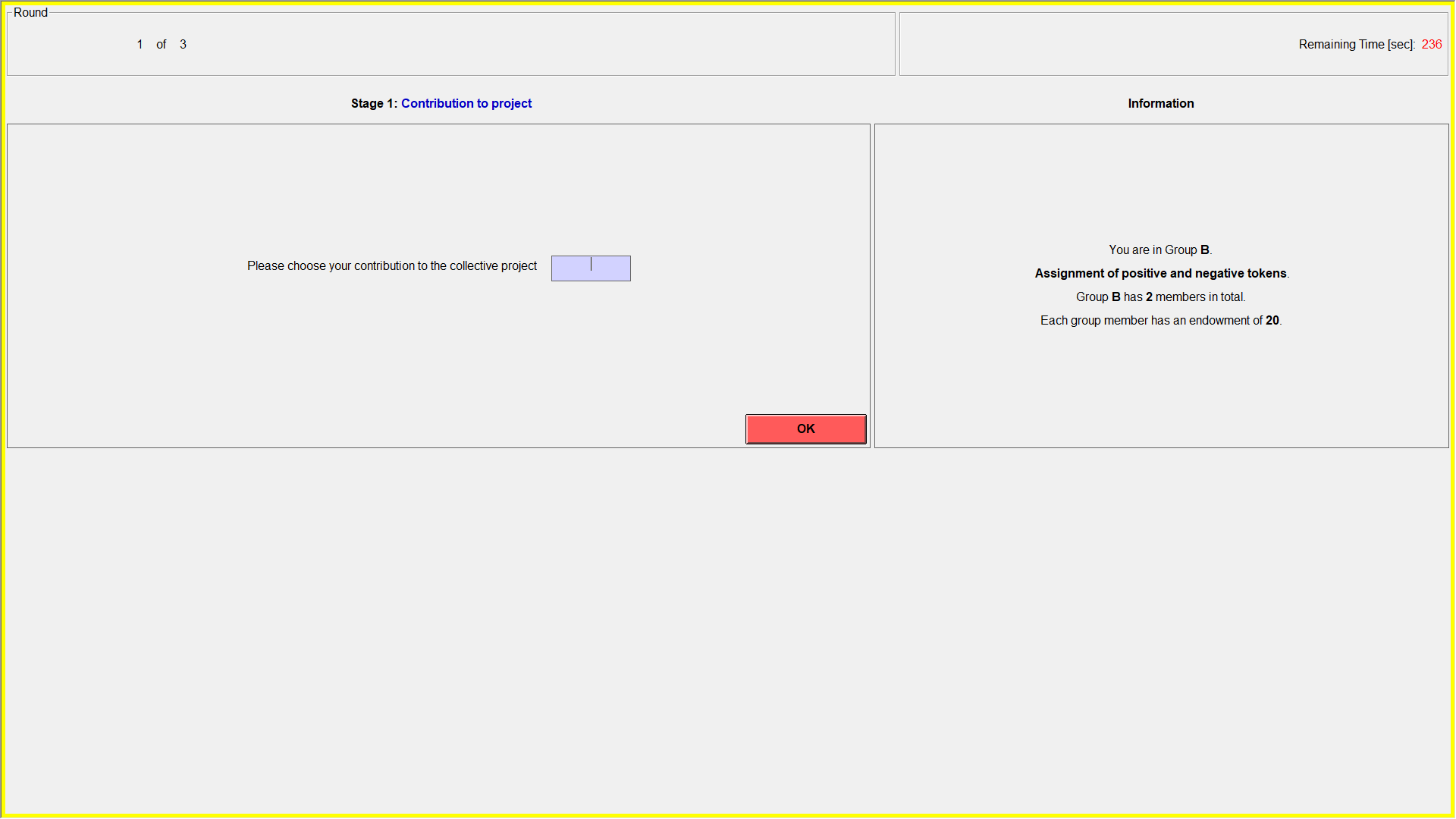
**Figure S3.**

This is again the “Stage 1: Contribution to project” screen. In this example you are in Group B, and you are one of two members in the group. Here you can decide how much you want to contribute to the collective project.


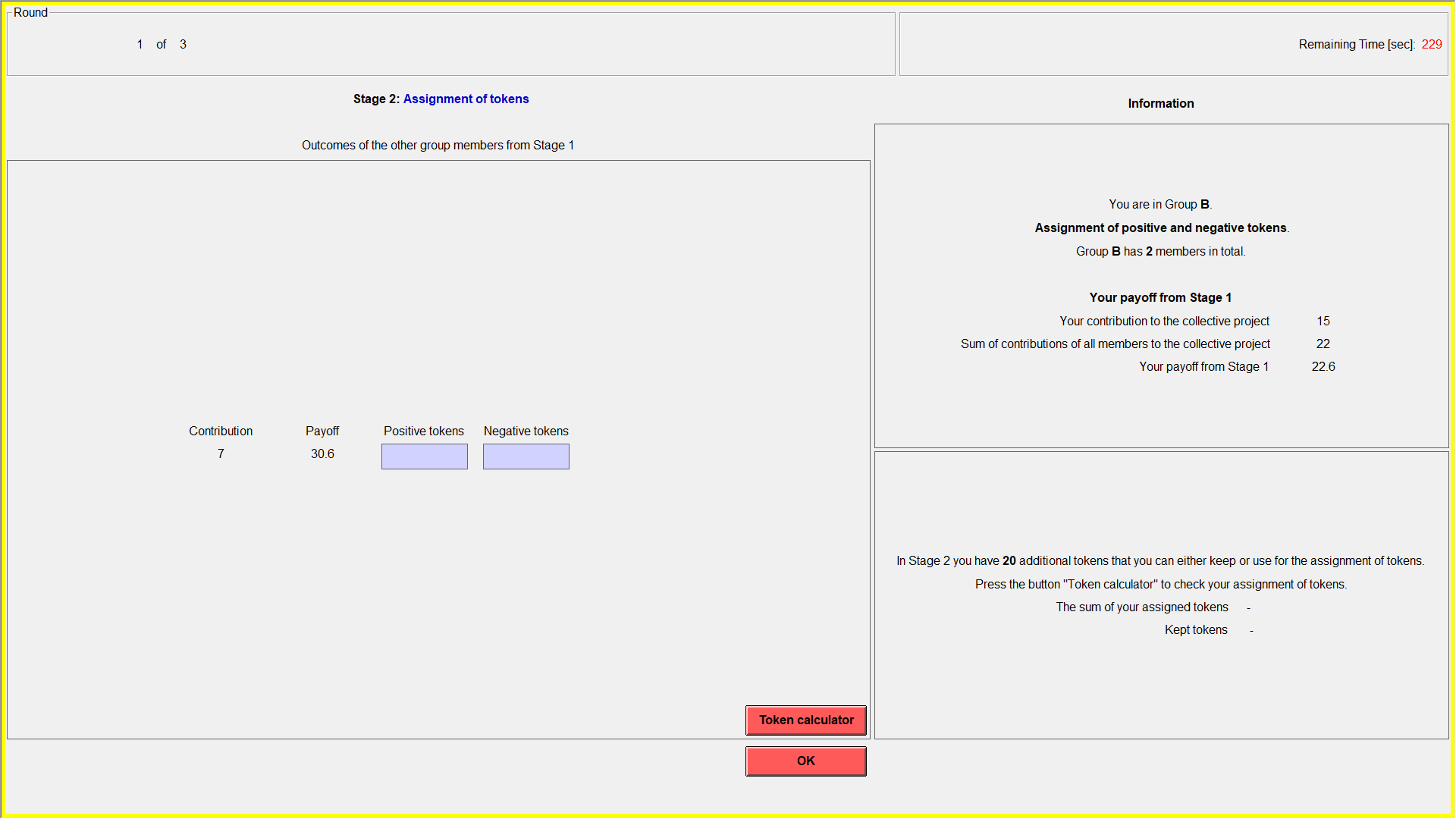
**Figure S4.**

This is the “Stage 2: Assignment of tokens” screen, which only appears if you are in Group B. In the central box to the left, you can see the contribution and the payoff of each group member (in this case there is only one other group member). Note that the order of presentation is randomly determined in each round and therefore it is not possible to identify any group member. In the central box you can decide how many of your additional 20 tokens you want to assign to other group members. By your assignment of tokens, you can increase or reduce the payoff of a group member or keep it unchanged. You can check the costs of your token assignment by pressing the button “Token calculator” before pressing “OK”. The output will be shown in the bottom-right corner. The information box on the right also indicates your payoff from Stage 1.


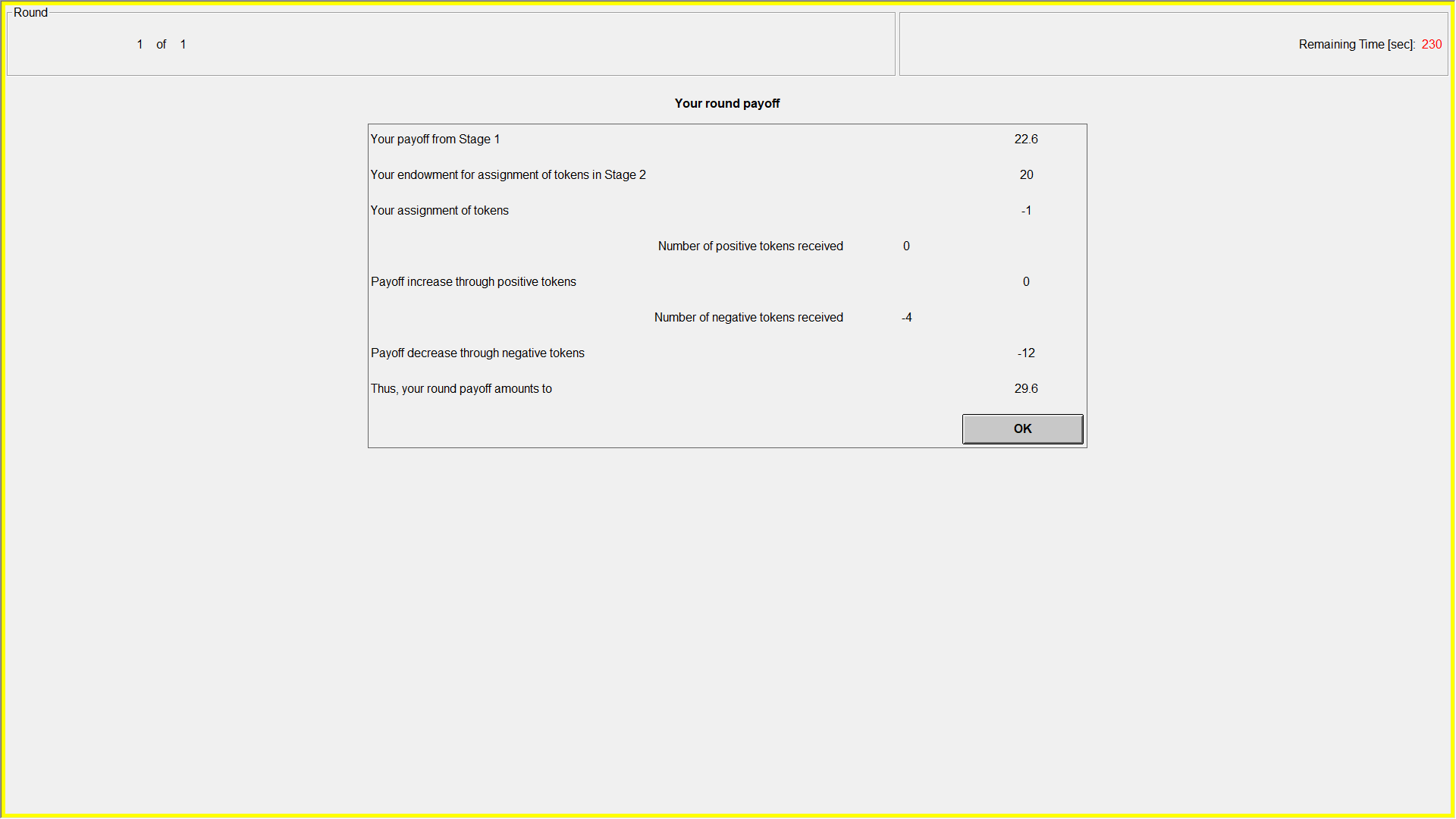
**Figure S5.**

This is “Your round payoff” screen. Here you can see how many positive and/or negative tokens you have received, your payoff in Stage 1 and 2, and your total round payoff.

In this example, the participant earned 22.6 tokens in Stage 1. In Stage 2, the participant received 20 additional tokens, assigned 1 token to another group member (i.e. -1), and received 0 positive tokens and 4 negative tokens from other group members. Since each negative token reduces the payoff of other group members by 3 tokens, the participant lost an additional 12 tokens. Thus, this participant’s total round payoff is 22.6 + 20 – 1 – 12 = 29.6 tokens.


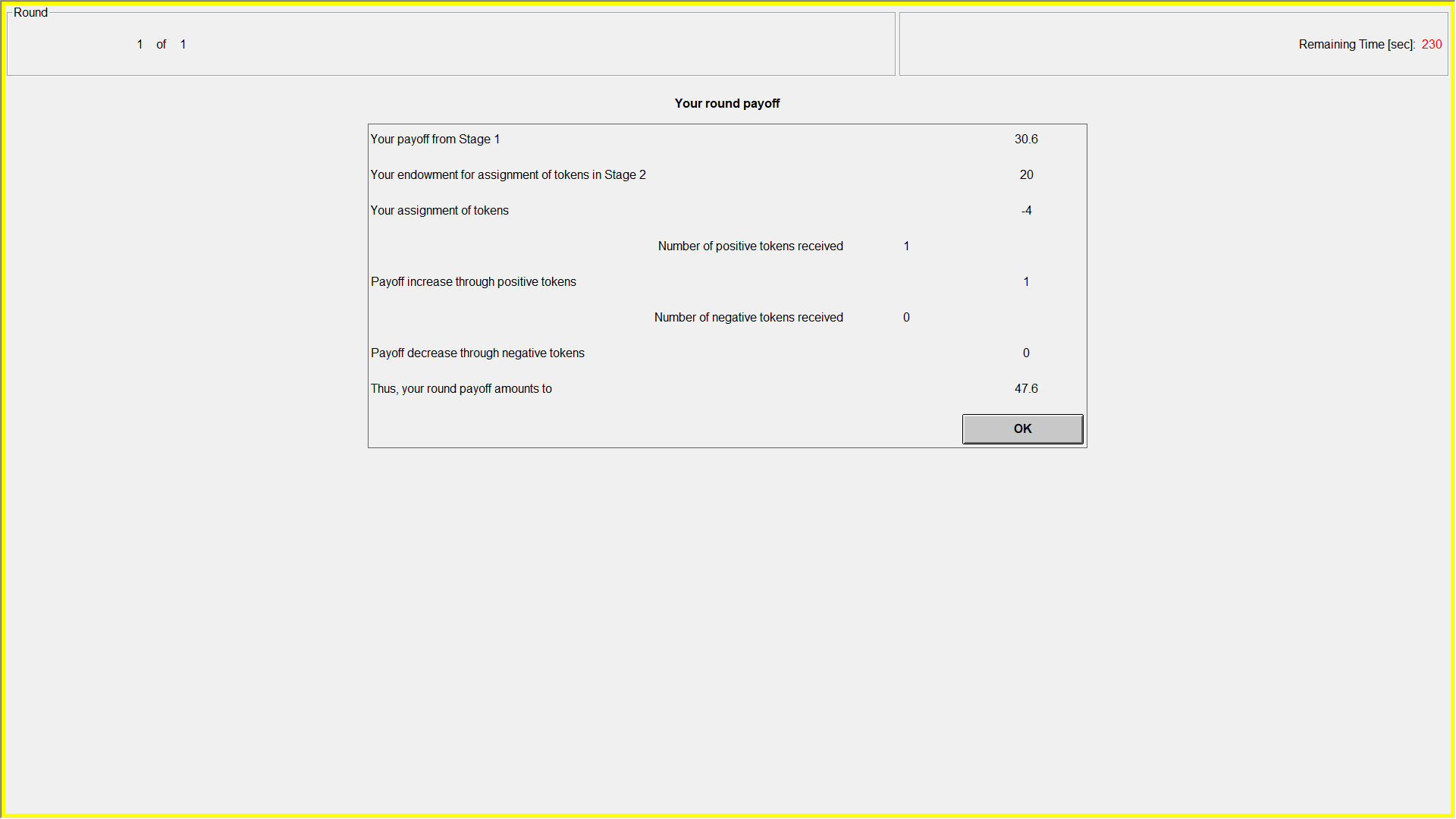
**Figure S6.**

This is again “Your round payoff” screen.

In this example, the participant earned 30.6 tokens in Stage 1. In Stage 2, the participant received 20 additional tokens, assigned 4 tokens to another group member (i.e. -4), and received 1 positive token and 0 negative tokens from other group members. Since each positive token increases the payoff of another group member by 1 token, the participant earned 1 additional token. Thus, this participant’s total round payoff is 30.6 + 20 – 4 + 1 = 47.6 tokens.


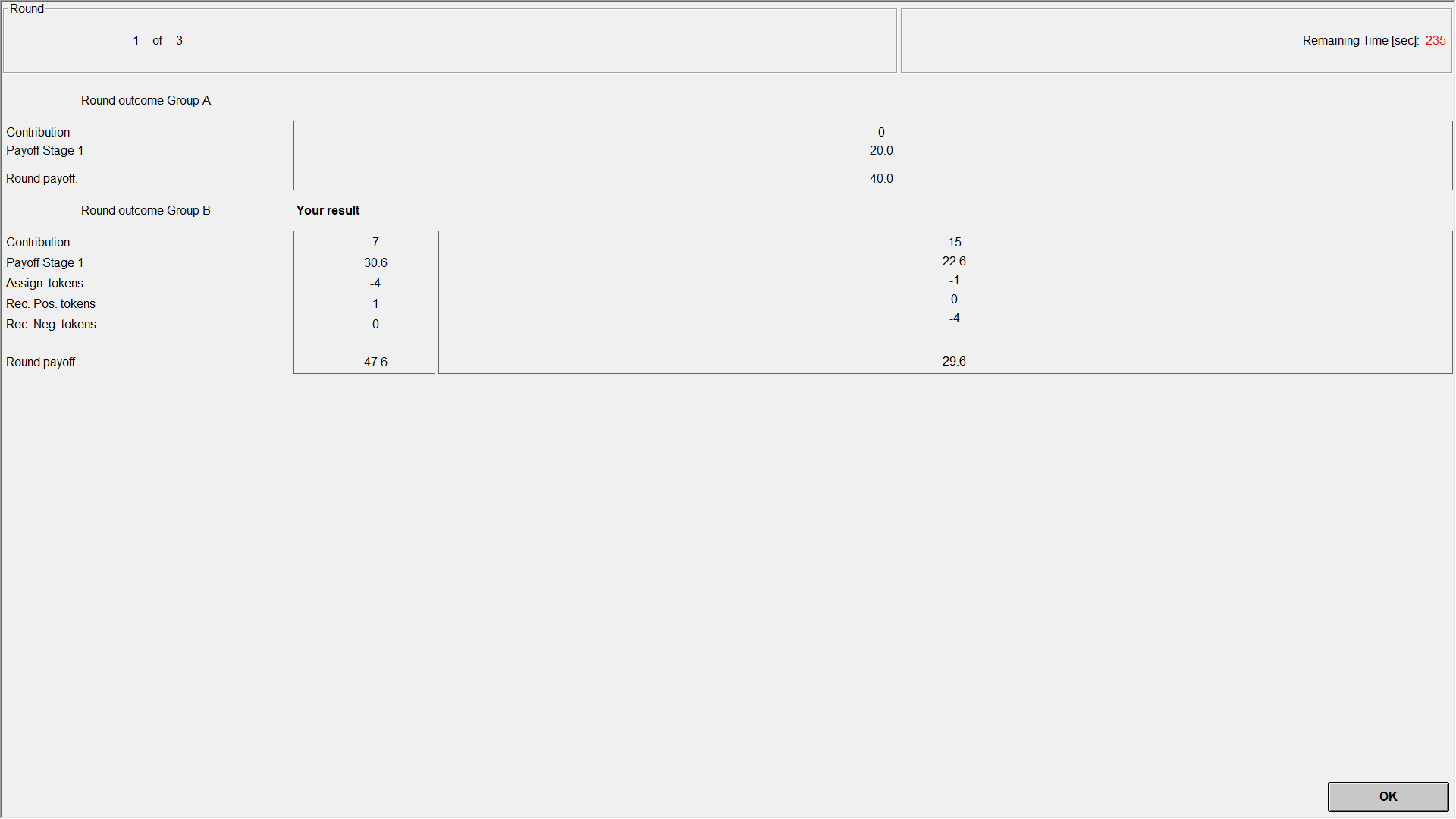
**Figure S7.**

This is the “Round Summary” screen.

At the end of the round you receive a detailed overview of the results obtained in both Groups. For every group member you are informed about their contribution to the collective project, payoff from Stage 1, assigned tokens (if applicable), received tokens (if applicable), and round payoff. Here you also see that the participant in Group A received 20 tokens in Stage 2 even though the participant could not use them to exercise influence on the outcome of other members of the group.


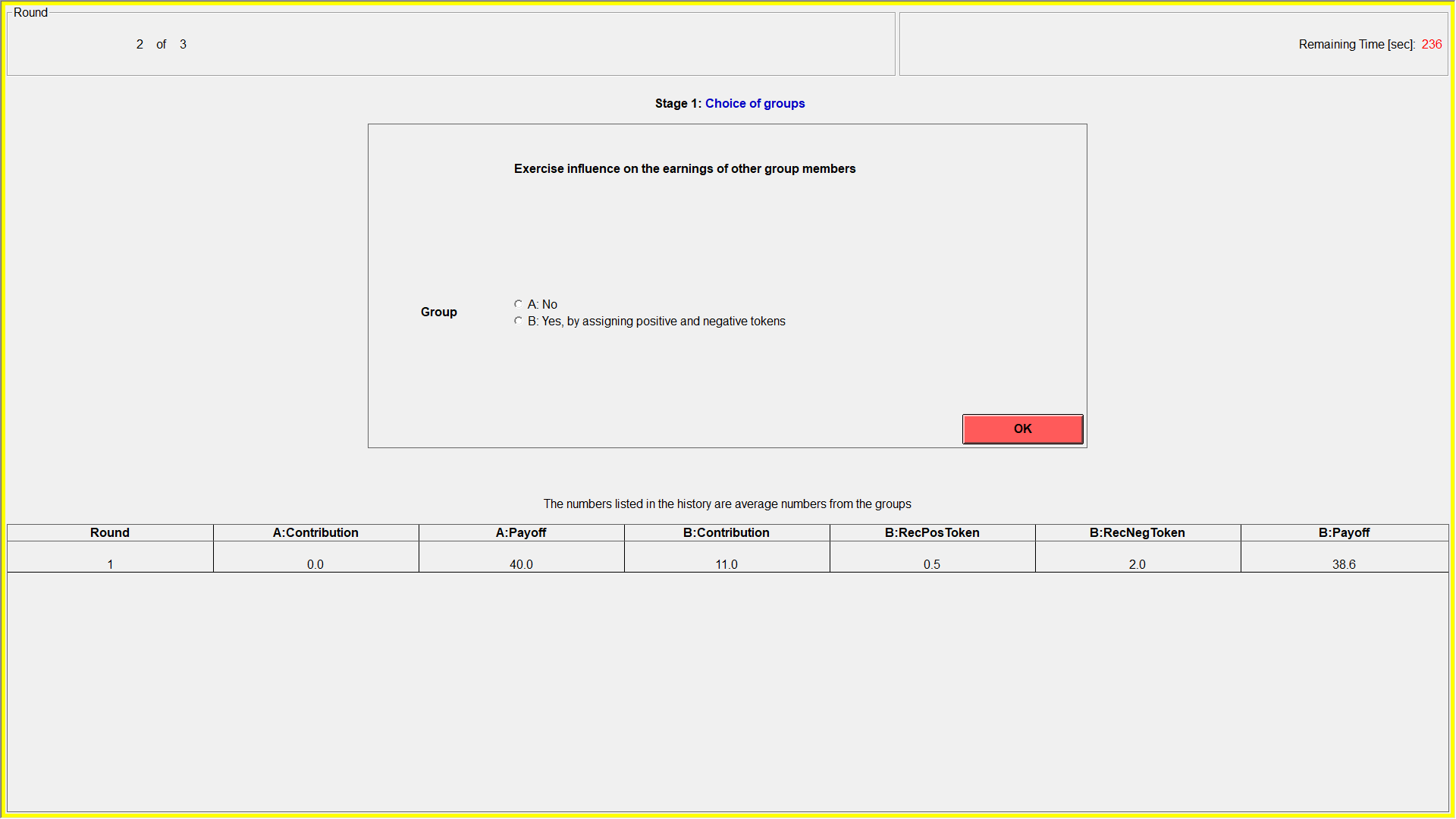
 **Figure S8.**

This is the “Stage 1: Choice of group” screen in Round 2. Here you can again choose in which group you want to be.

Starting with the 2^nd^ round you receive an overview of the average results of all previous rounds at the bottom of the screen. You can see the following information: average contribution of members of Group A, average payoff for members of Group A, average contribution of members of Group B, average positive tokens received for members of Group B, average negative tokens received for members of Group B, and the average payoff of members of Group B.

**Reference List**

Duch, Matthias L, Max RP Grossmann, and Thomas Lauer. 2020. "z-Tree unleashed: A novel client-integrating architecture for conducting z-Tree experiments over the Internet." Journal of Behavioral and Experimental Finance 28:100400.

van Aert, R. C. M., & Van Assen, M. A. L. M. 2017. “Bayesian evaluation of effect size after replicating an original study.” PLOS ONE, 12(4), e0175302. https://doi.org/10.1371/journal.pone.0175302

1. Using G*power 3.1.9.4, assuming a two-tailed Wilcox signed-rank test (α=.05) based on an underlying normal parent distribution. [↑](#footnote-ref-1)
